# Effect of the sonic hedgehog inhibitor GDC-0449 on an in vitro isogenic cellular model simulating odontogenic keratocysts

**DOI:** 10.1038/s41368-018-0034-x

**Published:** 2019-01-05

**Authors:** Jiemei Zhai, Heyu Zhang, Jianyun Zhang, Ran Zhang, Yingying Hong, Jiafei Qu, Feng Chen, Tiejun Li

**Affiliations:** 1 Department of Oral Pathology, Peking University School and Hospital of Stomatology, 22 South Zhongguancun Avenue, Haidian District Beijing, China; 2 Department of Oral Pathology, School of Stomatology Kunming Medical University, 1088 Middle Haiyuan Road, High-tech Zone, Kunming, China; 3 Central Laboratory, Peking University School and Hospital of Stomatology, 22 South Zhongguancun Avenue, Haidian District Beijing, China; 4 Peking University Hospital of Stomatology First Clinical Division, 37A Xishiku Street, Xicheng District, Beijing, China

## Abstract

Odontogenic keratocysts (OKCs) are common cystic lesions of odontogenic epithelial origin that can occur sporadically or in association with naevoid basal cell carcinoma syndrome (NBCCS). OKCs are locally aggressive, cause marked destruction of the jaw bones and have a propensity to recur. *PTCH1* mutations (at ∼80%) are frequently detected in the epithelia of both NBCCS-related and sporadic OKCs, suggesting that PTCH1 inactivation might constitutively activate sonic hedgehog (SHH) signalling and play a major role in disease pathogenesis. Thus, small molecule inhibitors of SHH signalling might represent a new treatment strategy for OKCs. However, studies on the molecular mechanisms associated with OKCs have been hampered by limited epithelial cell yields during OKC explant culture. Here, we constructed an isogenic *PTCH1*^R135X/+^ cellular model of *PTCH1* inactivation by introducing a heterozygous mutation, namely, c.403C>T (p.R135X), which has been identified in OKC patients, into a human embryonic stem cell line using the clustered regularly interspaced short palindromic repeats (CRISPR)/CRISPR-associated 9 (Cas9) system. This was followed by the induction of epithelial differentiation. Using this in vitro isogenic cellular model, we verified that the *PTCH1*^R135X/+^ heterozygous mutation causes ligand-independent activation of SHH signalling due to *PTCH1* haploinsufficiency. This activation was found to be downregulated in a dose-dependent manner by the SHH pathway inhibitor GDC-0449. In addition, through inhibition of activated SHH signalling, the enhanced proliferation observed in these induced cells was suppressed, suggesting that GDC-0449 might represent an effective inhibitor of the SHH pathway for use during OKC treatment.

## Oral health: repurposing existing tools to beat oral tumours

Using gene-edited cell cultures, Chinese researchers have gathered useful insights into the genetic origins and treatment of benign oral tumours called odontogenic keratocysts (OKCs). A total of 80% of OKCs are associated with mutations in the gene *PTCH1*, which are thought to activate a signalling pathway that drives OKC tumour growth. However, research is hindered by OKC cell culturing limitations. Tiejun Li, from the Peking University School and Hospital of Stomatology, and colleagues created an OKC cell culture model that uses CRISPR gene editing to introduce a *PTCH1* gene mutation into human stem cells before differentiating them into epithelial cells. Using their model, the team confirm that *PTCH1* mutation activates tumour-associated signalling, which the basal cell carcinoma drug vismodegib greatly reduces. Vismodegib also reduces the overproliferation of mutant cells and offers a potential treatment option for OKC patients.

## Introduction

Odontogenic keratocysts (OKCs), also known as keratocystic odontogenic tumours (KCOTs), are common odontogenic lesions that can occur sporadically or in association with naevoid basal cell carcinoma syndrome (NBCCS). They are locally aggressive, can cause marked destruction of the jaw bones and have a propensity for recurrence after surgery due to enhanced proliferation in epithelial linings compared with other odontogenic cysts, such as dentigerous cysts and radicular cysts.^1–6^ Although the World Health Organization resumed the original terminology, “OKC”, in its new classification, uncertainty remains over the nature of this odontogenic lesion.

NBCCS is a rare autosomal dominant disease characterized by developmental abnormalities and a predisposition to various tumours, such as multiple basal cell carcinomas (BCCs), medulloblastoma and rhabdomyosarcoma.^7,8^ *PTCH1*, which is homologous to *Drosophila patched*, acts as a tumour suppressor and encodes a putative signal transducer of sonic hedgehog protein (SHH). It is well known that inactivation of PTCH1 causes NBCCS.^9,10^ Binding of SHH to PTCH1 or mutational inactivation of PTCH1 relieves the inhibition of SMO, a G protein-coupled receptor-like protein, which might result in SHH pathway activation in a ligand-dependent or ligand-independent manner, respectively. Consequently, this results in the expression of target genes such as *GLI1* and *PTCH1*, leading to developmental abnormalities and tumourigenesis. PTCH1 is both a receptor of the SHH ligand and a target gene of the SHH pathway, forming a negative feedback mechanism that regulates the pathway, thus it is difficult to predict SHH pathway activation based on *PTCH1* expression. However, *GLI1* mRNA levels are a reliable indicator of SHH pathway activation.^11–13^

Our previous studies showed frequent heterozygous mutations of *PTCH1* (~80%) in the epithelia of both NBCCS-related and sporadic OKCs, most of which were expected to result in the synthesis of truncated PTCH1 proteins.^14,15^ According to the model of SHH signalling, we postulated that frequent *PTCH1* mutations in OKCs might cause ligand-independent activation of SHH signalling and play a major role in the pathogenesis of this disease. Thus, small molecules inhibiting the SHH signalling pathway might represent new treatment strategies. Recent evidence suggests that GDC-0449 (vismodegib), an FDA-approved SMO inhibitor for the treatment of locally advanced and metastatic BCCs, appears to be effective in treating OKCs. Following treatment with this agent, some syndromic OKCs were found to decrease in size with a corresponding increase in sclerosis at the site of prior lesions.^16–19^ However, a basic understanding of molecular-targeted therapies has been hampered by limited epithelial cell yields from primary OKC cultures from fresh tissues.

Human embryonic stem cells (hESCs), capable of self-replication and unlimited proliferation, can differentiate into various somatic cell types derived from all three germ layers with a stable diploid karyotype. Thus, hESCs are considered a source of unlimited cells for disease modelling and drug screening.^20,21^ Recent advances in gene editing using hESCs have facilitated the generation of patient/disease-specific cell models in vitro.^22,23^ In this study, we established an in vitro isogenic *PTCH1*^R135X/+^ cellular model to accurately model the genetics of OKC by introducing a *PTCH1* heterozygous truncating mutation, specifically c.403C>T (p.R135X), which was found in OKC patients, into hESCs using the clustered regularly interspaced short palindromic repeats (CRISPR)/CRISPR-associated 9 (Cas9) gene editing technique. Targeted hESCs were then differentiated into epithelial progenitor cells (hESC-Es). The status of SHH signalling and the proliferative ability of this cell model were then investigated in vitro. GDC-0449, a small molecule inhibitor of the SHH pathway, was tested for its effects on SHH signalling and cell proliferation in these induced hESC-Es. Thus, this study was designed to provide further insight into the molecular mechanisms associated with OKCs and yield new molecular strategies to treat this disease.

## Results

### Construction of PTCH1^R135X/+^ hESCs

**Fig. 1:**
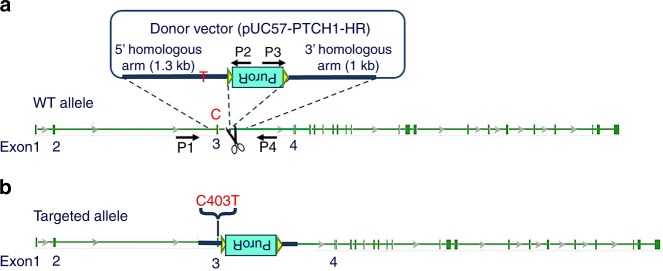
Schematic representation of the CRISPR targeting strategy. **a** A c.403C>T mutation was introduced into exon 3 of *PTCH1*. The scissors represent Cas9/sgRNA targeting the sequence of intron 3 near the mutation site, resulting in DNA double-stranded breaks. The donor vector contained the inverted drug selection cassette PuroR, flanked by a 1.3-kb 5′ homologous arm carrying the c.403C>T point mutation and a 1-kb 3′ homologous arm. **b** The c.403C>T point mutation was predicted to be knocked into the hESC genome by homologous recombination. Primers P1, P2, P3 and P4 were used to screen the positive clones

**Fig. 2:**
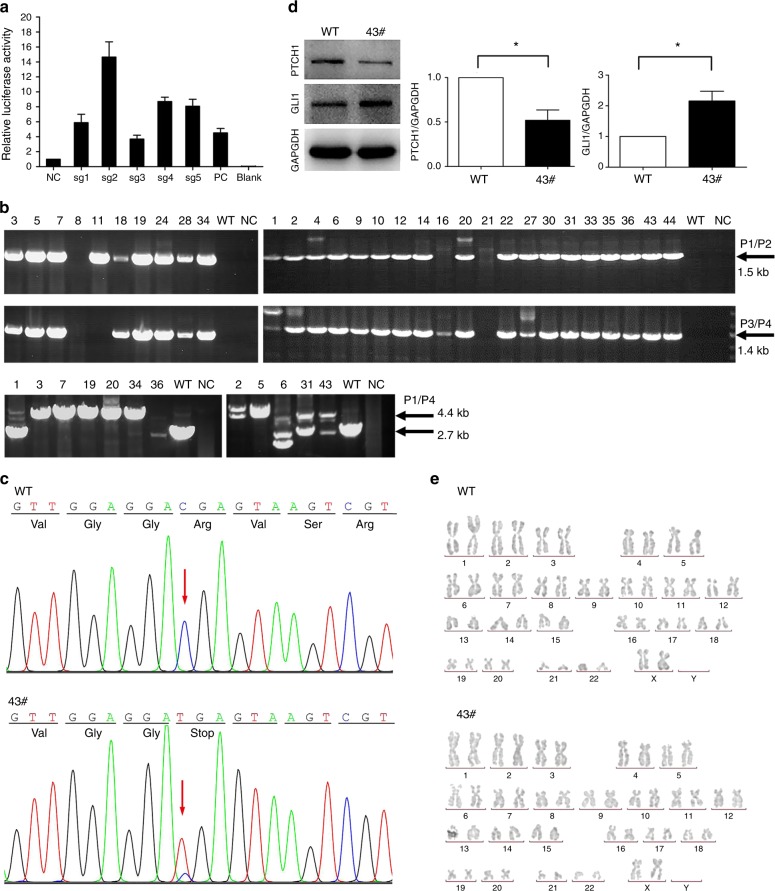
Site-directed mutagenesis of *PTCH1* using the CRISPR/Cas9 system. **a** Cleavage activity of the Cas9/sgRNA plasmid in HEK293T cells. sgRNA-2 was chosen for gene targeting. **b** Thirty drug-resistant clones were identified by 5′-junction PCR (P1/ P2, 1.5 kb) and 3′-junction PCR (P3/ P4, 1.4 kb). Twelve positive clones were further identified by long-range PCR (P1/P4, 4.4 kb or 2.7 kb). **c** Sequencing of the c.403C>T mutation site in clone 43 hESCs using primer pair PTCH1-F/PTCH1-R. Wild-type human embryonic stem cells (WT-hESCs) were used as the control. Clone 43 showed a heterozygous C/T peak indicating that one allele was successfully mutated. **d** Western blotting revealed a 50% decrease in PTCH1 protein expression and a twofold increase in GLI1 expression compared with WT-hESCs (*n* = 3, *P < 0.05). **e** Karyotype analysis showed that both WT hESCs and clone 43 hESCs retained a normal diploid chromosomal karyotype (46, XX)

The targeting strategy is summarized in Fig. 1. Since the c.403C>T mutation resides in exon 3 of *PTCH1*, five specific single-guide RNAs (sgRNAs) of ~20 bp, designed herein, were used to target intron 3, near the mutation (Table 1). In this study, sgRNA-2 was chosen for gene targeting, as its relative cleavage activity was up to 14-fold and 3-fold greater than that of the negative control (NC) and positive control (PC), respectively (Fig. 2a). After gene targeting, 30 puromycin-resistant clones were picked and expanded in 48-well plates; these were then seeded in 12-well plates for cryopreservation and genotype identification, from which 12 clones were selected and further assessed by long-range PCR (Fig. 2b). Finally, clone 43 was characterized as a *PTCH1* c.403C>T heterozygous mutant based on DNA sequencing (Fig. 2c) and was selected for downstream experiments. Western blotting confirmed successful gene editing, revealing a near 50% decrease in PTCH1 protein expression and twofold increase in GLI1 expression compared with wild-type (WT) hESCs (Fig. 2d). Karyotype analysis demonstrated that clone 43 retained a normal 46XX karyotype (Fig. 2e).

**Table 1:** sgRNA sets designed to target the *PTCH1* locus

	sgRNA	Sequence (5′- 3′)	Extra base	Target sequence length
PTCH1-sgRNA1	Target sequence	CACTTGGGGACTATACACCCAGG	1	21 bp + PAM
	PTCH1-sgRNA1-up	*cacc***G**CACTTGGGGACTATACACCC		
	PTCH1-sgRNA1-dn	*aaac*GGGTGTATAGTCCCCAAGTG**C**		
PTCH1-sgRNA2	Target sequence	CTGGGTGTATAGTCCCCAAGTGG	1	21 bp + PAM
	PTCH1-sgRNA2-up	*cacc***G**CTGGGTGTATAGTCCCCAAG		
	PTCH1-sgRNA2-dn	*aaac*CTTGGGGACTATACACCCAG**C**		
PTCH1-sgRNA3	Target sequence	GGATGACGCTGAGCCACTTGGGG	0	20 bp + PAM
	PTCH1-sgRNA3-up	*cacc*GGATGACGCTGAGCCACTTG		
	PTCH1-sgRNA3-dn	*aaac*CAAGTGGCTCAGCGTCATCC		
PTCH1-sgRNA4	Target sequence	GTGTCACACAGCACGTTGGGGG	0	20 bp + PAM
	PTCH1--sgRNA4-up	*cacc*GTGTCACACAGCACGTTGG		
	PTCH1--sgRNA4-dn	*aaac*CCAACGTGCTGTGTGACAC		
PTCH1-sgRNA5	Target sequence	GTTTTGTGTCACACAGCACGTTGG	0	20 bp + PAM
	PTCH1-sgRNA5-up	*cacc*GTTTTGTGTCACACAGCACGT		
	PTCH1-sgRNA5-dn	*aaac*ACGTGCTGTGTGACACAAAAC		

Underlined capital letters represent protospacer adjacent motifs (PAM) in the target sequence. Italic lowercase letters represent the sticky ends after annealing. Bold capital “G/C” represents an additional “G/C” prepended for the expression of sgRNAs from a U6 promoter

### Differentiation of WT and mutant hESCs into hESC-Es

**Fig. 3:**
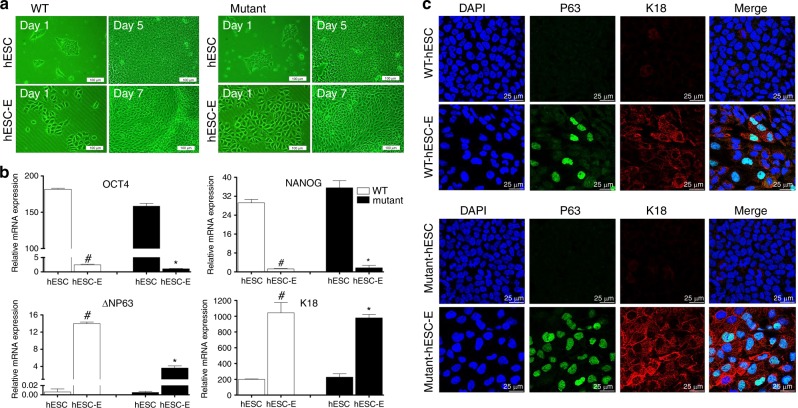
Direct differentiation of human embryonic stem cells (hESCs) into epithelial progenitor cells (hESC-Es). **a** Morphological changes in wild-type (WT) (*PTCH1*^+/+^) and mutant (*PTCH1*^R135X/+^) hESCs before and after epithelial induction, as assessed by inverted phase contrast microscopy. Scale bar = 100 μm. **b** Quantitative real-time PCR analysis of the expression of pluripotent markers (*OCT4*, *NANOG*) and keratinized epithelial markers (*K18*, *ΔNP63*) in undifferentiated hESCs and induced hESC-Es. Data represent the mean ± SD, *n* = 3 (#, WT hESC-Es vs WT hESCs; *, mutant hESC-Es vs mutant hESCs; #, *, *P* < 0.05). **c** Immunofluorescence images of P63 (green) and K18 (red) expression in WT and mutant cells before and after induction. The left panel displays corresponding DAPI nuclear staining (blue) and the right panel displays corresponding merged images. Scale bar = 25 μm

To model the diseased epithelial cells, we used a direct differentiation protocol to induce differentiation of hESCs into hESC-Es. Similar to WT (*PTCH1*^+/+^) hESC-Es, mutant (*PTCH1*^R135X/+^) hESC-Es displayed a typical epithelial morphology with a polygonal shape and cobblestone-like arrangement during the 7 days of induction, whereas WT and mutant hESCs both maintained an undifferentiated state, with a high nucleus-to-cytoplasm ratio and similar colony morphology (Fig. 3a).

Previous studies have shown that hESC-Es can express the epithelial precursor cell markers P63 and K18 after epithelial induction for 7 d.^24,25^ The *P63* gene encodes the full-length transactivating (TA) and N-terminal truncated (ΔN) isotypes, among which ΔNP63 is the predominant isotype during epithelial differentiation.^26^ Consistent with published data, real-time PCR demonstrated that after epithelial induction for 7 d, the expression levels of pluripotency markers *OCT4* and *NANOG* were significantly downregulated, whereas the expression levels of epithelial differentiation markers *ΔNP63* and *K18* were significantly upregulated compared with undifferentiated hESCs (Fig. 3b). Immunofluorescence double-staining of hESC-Es further confirmed that both WT and mutant hESCs were able to differentiate into epithelial progenitor cells at day 7, as shown by positive expression of P63 (green, observed in the nucleus) and K18 (red, expressed in the cytoplasm and capsule). Furthermore, in some cellular regions, co-expression of P63 and K18 was observed, highlighted in Fig. 3c. Undifferentiated WT and mutant hESCs served as control groups.

### Status of SHH signalling in WT and mutant hESC-Es

**Fig. 4:**
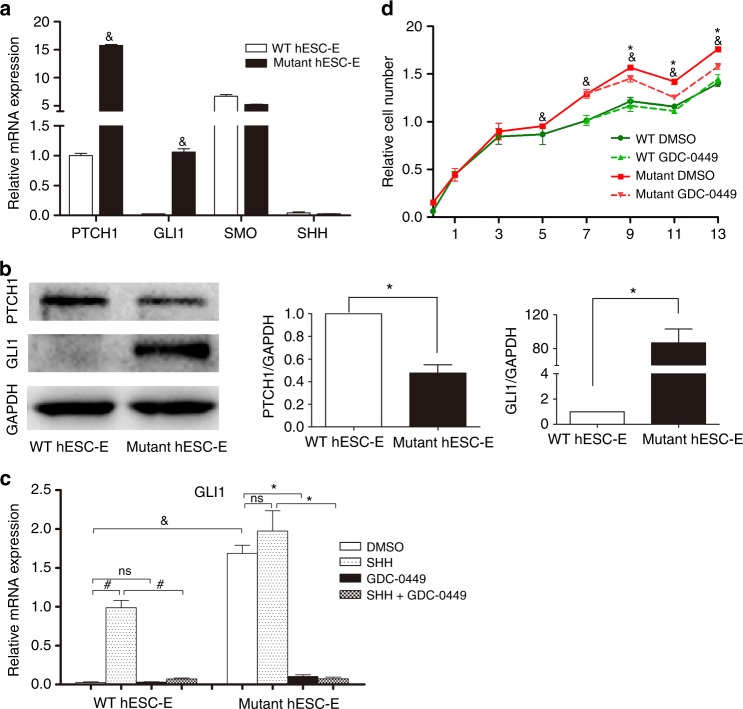
Status of sonic hedgehog (SHH) signalling and effects of GDC-0449 on wild-type (WT) and mutant human epithelial progenitor cells (hESC-Es) in vitro. **a** Real-time PCR demonstrating the mRNA levels of components of the SHH signalling pathway (*PTCH1*, *GLI1*, *SMO* and *SHH*) in WT (*PTCH1*^+/+^) and mutant (*PTCH1*^R135X/+^) hESC-Es. Data represent mean ± SD, *n* = 3 (&, mutant hESC-Es vs WT hESC-Es, *p* < 0.05). **b** Western blotting showed the protein levels of SHH pathway target genes (*PTCH1* and *GLI1*) in WT and mutant hESC-Es. Expression of PTCH1 was reduced by half, and expression of GLI1 was significantly upregulated in mutant hESC-Es compared with WT hESC-Es (Data represent mean ± SD, *n* = 3, *P < 0.05). **c** The relative mRNA expression of *GLI1* with exogenous SHH (1 μg•mL^-1^) and/or GDC-0449 (1 μmol•L^-1^) treatment for 24 h (&, #, *, *P* < 0.05; ns, *P* > 0.05). **d** GDC-0449 (1 μmol•L^-1^) was tested for its effects on the cell proliferation of WT and mutant hESC-Es. WT and mutant DMSO vehicle groups served as respective controls (&, mutant control group vs WT control group; *, mutant GDC-0449-treated group vs mutant DMSO control group; &, *, *P* < 0.05)

To investigate the status of SHH signalling in *PTCH1*^R135X/+^ hESC-Es, we analyzed the mRNA levels of key components of the SHH signalling pathway (specifically, *PTCH1*, *GLI1*, *SMO* and *SHH*) by real-time PCR (Fig. 4a), among which *GLI1* expression is a reliable indicator of SHH pathway activity.^13,27^ The mRNA levels of the SHH target genes *PTCH1* and *GLI1* were significantly upregulated by 15.8-fold and 43.1-fold, respectively, in mutant hESC-Es compared with those in WT hESC-Es. The high expression of *GLI1* suggested that SHH signalling was highly activated in mutant hESC-Es. In contrast, the mRNA levels of *SMO* and *SHH* were not significantly changed when compared with those in WT hESC-Es. In addition, the low expression of *SHH* indicated that the SHH ligand might not be necessary for activation of this signalling pathway in mutant hESC-Es, which was further confirmed by subsequent experiments.

Furthermore, the protein levels of SHH target genes, namely, *PTCH1* and *GLI1*, were analyzed in hESC-Es by western blotting. The significant upregulation of GLI1 in mutant hESC-Es was consistent with that observed at the mRNA level, confirming the abnormal activation of SHH signalling. However, although *PTCH1* mRNA levels increased, PTCH1 protein expression was decreased, as expected, in mutant hESC-Es compared with that in the WT hESC-Es (Fig. 4b). It has been reported that loss of PTCH1 function by inactivating mutations can cause SHH ligand-independent activation of the SHH pathway, leading to neoplastic growth.^28,29^ Our data suggested that overexpression of the *PTCH1* transcript occurred predominantly from the mutated allele carrying the c.403C>T (p.R135X) mutation, which was expected to encode a truncated (nonfunctional) PTCH1 protein (of only 135 amino acids). Despite the presence of one WT allele, the heterozygous c.403C>T (p.R135X) truncating mutation of one *PTCH1* allele, resulting in the reduction of full-length (functional) PTCH1 protein expression, also known as haploinsufficiency, could have been the cause of activated SHH signalling in *PTCH1*^R135X/+^ hESC-Es.

### Effects of GDC-0449 on WT and mutant hESC-Es

Having detected that SHH pathway signalling is highly activated in *PTCH1*^R135X/+^ hESC-Es, we next tested the effects of exogenous recombinant human SHH and/or an SHH pathway inhibitor, GDC-0449, on these cells. To determine the optimal concentration of GDC-0449, induced hESC-Es at day 7 were treated with different concentrations (0.01, 0.1, 1 and 10 μmol•L^-1^) of GDC-0449 or dimethylsulfoxide (DMSO, vehicle control) for 24 h. Total RNA was extracted and real-time PCR analysis of SHH target genes was performed. The results showed that WT hESC-Es were insensitive to GDC-0449 based on *GLI1* expression. However, SHH signalling was highly activated and could be downregulated in a dose-dependent manner with the addition of GDC-0449 to mutant hESC-Es; specifically, 1 µmol•L^-1^ was the smallest dose that resulted in maximum inhibition (Fig. S1) and was thus selected for the subsequent experiments. The concentration of recombinant human SHH (1 μg•mL^-1^, GenScript) to use was decided based on previous studies.^30,31^

The mRNA expression of *GLI1* with or without drug treatment is shown in Fig. 4c. SHH signalling was highly activated in *PTCH1*^R135X/+^ hESC-Es, as *GLI1* mRNA was significantly upregulated compared with expression in WT hESC-Es (*P* < 0.05). In WT hESC-Es, the expression of *GLI1* was significantly upregulated by 39-fold *(P* < 0.05) after treatment with 1 μg•mL^-1^ of exogenous SHH when compared with the expression in the DMSO vehicle group. In addition, SHH-induced upregulation of this transcript could be suppressed to control the levels with 1 μmol•L^-1^ GDC-0449 (*P* < 0.05). However, no significant difference was observed with 1 μmol•L^-1^ GDC-0449 treatment alone in WT hESC-Es (*P* > 0.05). In contrast, mutant hESC-Es responded marginally to 1 μg•mL^-1^ of exogenous SHH, as measured by *GLI1* mRNA levels (*P* > 0.05), and this change was significantly suppressed with 1 μmol•L^-1^ GDC-0449 to levels far lower than those in the untreated group (*P* < 0.05). Meanwhile, 1 μmol•L^-1^ GDC-0449 alone markedly inhibited *GLI1* expression in mutant hESC-Es compared with the DMSO vehicle group (*P* < 0.05), with an observed inhibition of 93.9%. Therefore, the results further confirmed that activation of the SHH pathway in *PTCH1*^R135X/+^ hESC-Es is SHH ligand-independent and that GDC-0449 inhibits activated SHH signalling in a dose-dependent manner in *PTCH1*^R135X/+^ hESC-Es.

In addition, the effect of GDC-0449 on cell proliferation was determined by performing a cell counting kit-8 (CCK-8) assay. After day 5 of differentiation, we observed that the total number of *PTCH1*^R135X/+^ cells was higher than that of WT induced cells (*P* < 0.05), suggesting that *PTCH1*^R135X/+^ hESC-Es have increased proliferative ability; this could be suppressed through treatment with 1 µmol•L^-1^ GDC-0449 (*P* < 0.05). However, 1 μmol•L^-1^ GDC-0449 had no apparent effect on cell proliferation in WT hESC-Es, as shown in Fig. 4d. These data indicate a link between SHH signalling status and cell proliferation.

## Discussion

More than 200 *PTCH1* mutations have been detected in syndromic and sporadic OKCs, most of which were the predicted truncation-causing mutations resulting in partial loss of PTCH1 expression. These detected mutations were mostly scattered across the *PTCH1* gene with no apparent hotspot.^14,15^ Only occasional recurrent mutations in *PTCH1* were reported in OKCs.^15,32,33^ Here, we established an isogenic *PTCH1*^R135X/+^ cellular model. The c.403C>T heterozygous point mutation was detected in OKCs from three unrelated NBCCS patients.^32,33^ It is predicted to cause a truncation in exon 3, resulting in a premature stop at codon 135 in the first-predicted extracellular loop of PTCH1, which is a hot region for mutations associated with syndromic and sporadic OKCs.^15^ Therefore, this mutation represents one of the occasionally recurrent *PTCH1* mutations encountered in OKC, which is why it was selected for targeting.

Three gene-targeting techniques have been developed based on DNA double-stranded breaks, which subsequently stimulate endogenous DNA repair machinery through homologous recombination or non-homologous end joining.^34^ Compared to the zinc-finger nuclease and transcription activator-like effector nuclease technique, the CRISPR/Cas9 system is associated with higher efficiency and easier construction; the simple design of an sgRNA, of ∼20 bp, has proven to be an efficient tool for gene editing.^35–38^ Here, we successfully knocked in the c.403C>T point mutation into one allele in WT hESCs, generating a *PTCH1*^R135X/+^ model (*PTCH1*^R135X/+^ hESCs) using the CRISPR/Cas9 system and a donor plasmid via homologous recombination.

To model the diseased epithelial cells, we then induced *PTCH1*^R135X/+^ hESCs to differentiate into epithelial progenitor cells (*PTCH1*^R135X/+^ hESC-Es). Metallo et al.^25^ demonstrated that a direct differentiation method eliminating the embryoid body formation process can provide a more uniform microenvironment during differentiation and give rise to higher purity and yield of p63+/K18+ epithelial progenitor cells.^24^ Using the direct differentiation method and retinoic acid-conditioned medium, we found that, similar to WT *PTCH1*^+/+^ hESCs, mutant *PTCH1*^R135X/+^ hESCs were able to differentiate into epithelial progenitor cells, as shown by their polygonal cobblestone-like cell morphology and positive expression of K18 and P63 at the mRNA and protein level. However, both WT and mutant hESC-Es survived poorly after passaging. Consequently, the hESC-Es used in the following experiments were obtained by continuous induction for only 7 days, and both WT and mutant hESCs were not differentiated into a more mature stage of epithelial cells. Future studies to improve the cell culture conditions need to be performed.

Using this cellular model, we discovered a ligand-independent mechanism of SHH pathway activation in *PTCH1*^R135X/+^ hESC-Es, as evidenced by the low expression of endogenous *SHH* and the lack of response to exogenous SHH. Previous studies on rhabdomyosarcoma using *PTCH1*^+/−^ mice reported the overexpression of mutated *PTCH1* transcripts; however, transcripts from the WT allele appeared to be downregulated, resulting in a reduction in functional PTCH1 protein expression. This suggests that haploinsufficiency of *Ptch1* is sufficient for rhabdomyosarcoma formation in a murine model.^39,40^ Our western blot results indicated that the heterozygous c.403C>T (p.R135X) truncating mutation in *PTCH1*, resulting in *PTCH1* haploinsufficiency, might also be sufficient to promote OKC development, possibly due to loss of negative feedback from *PTCH1*.

We also observed a link between SHH status and cell proliferation. Previous studies have shown that the epithelial lining of OKCs has increased proliferation potential compared with that of other types of odontogenic cysts, i.e. dentigerous cysts and radicular cysts.^3,5^ Particularly, those with *PTCH1* truncation-causing mutations had higher proliferative activity than OKCs with non-truncating mutations or WT *PTCH1*, based on immunohistochemical staining for Ki67 and/or PCNA.^41^ In this study, we verified that *PTCH1*^R135X/+^ cells have increased proliferative ability, as shown by CCK-8 assays using the *PTCH1*^R135X/+^ hESC-Es model. Furthermore, inhibiting activated SHH signalling in *PTCH1*^R135X/+^ hESC-Es with GDC-0449 resulted in decreased cell proliferation, suggesting that this drug could be used to target the SHH pathway for OKC treatment. The molecular mechanisms underlying SHH signalling and enhanced cell proliferation in *PTCH1*^R135X/+^ hESC-Es require further investigation.

In summary, an in vitro isogeneic cellular model simulating OKC was established using the CRISPR/Cas9 gene editing technique and the induction of epithelial differentiation. Our data provide evidence that SHH signalling is active in *PTCH1*^R135X/+^ hESC-Es. Inhibiting activated SHH signalling in *PTCH1*^R135X/+^ hESC-Es decreased cell proliferation, suggesting that GDC-0449 might be an effective candidate for targeting the SHH pathway during OKC treatment. However, the nondetection of *PTCH1* mutations in a subset of OKCs (∼20%)^14,15^ also underlines the nonexploration of other genetic events that may not be responsive to SHH inhibitors. Further, the known adverse effects of GDC-0449, such as alopecia, gastrointestinal and muscle spasms and dysgeusia, may also restrict its oral use. The so-called marsupialization method, involving the creation of a surgical window in the jaw to decompress intracystic pressure for a gradual reduction in cyst size, has become a common treatment for OKCs. This certainly raises the possibility of intracystic injection of SHH inhibitors as an alternative option. Taken together, our study provides an in vitro isogeneic cellular model for studying the pathogenesis of OKC, predicting the efficacy of molecular-targeted therapies and for further research to test novel therapeutics.

## Materials and methods

### Cell culture and epithelial differentiation

The hESC cell line H9 (Wicell, USA) was generously provided by Professor Tong Cao, National University of Singapore. Undifferentiated hESCs were cultured in an Essential 8 (E8, Invitrogen, USA) feeder-free culture system on Matrigel (BD Biosciences, San Jose, CA, USA)-coated plates. Cells were routinely passaged every 4 to 5 days when clones reached 75%–85% confluency, as previously described.^42^ Briefly, hESCs were washed in Dulbecco’s phosphate-buffered saline (DPBS, Invitrogen, USA) without calcium and magnesium and then treated with 1 mL per well of pre-warmed 0.5 mmol•L^-1^ ethylene diamine tetraacetic acid (EDTA, Invitrogen, USA) for ∼3–5 min at 37 °C. The EDTA was aspirated, cells were gently washed with E8 medium and cells were then split at an appropriate ratio. Cells were incubated at 37 °C in a 5% CO_2_ incubator overnight. E8 medium was changed daily.

To induce the differentiation of hESCs into hESC-Es, we used a direct differentiation method as described previously.^24,25^ In brief, hESCs were separated into single cells using TrypLE (Invitrogen, USA) and seeded on Matrigel-coated plates at a density of 6 500 cells per cm^2^ in E8 medium containing the ROCK inhibitor Y-27632 (Selleck Chemicals, Houston, TX, USA) at 10 μmol•L^-1^. The culture medium was switched to epithelial differentiation medium, consisting of DMEM/F12 (Invitrogen, USA) supplemented with 1 μmol•L^-1^ retinoic acid (Sigma, USA), 1× N2 supplement (Invitrogen, USA) and 25 ng•mL^-1^ bone morphogenic protein 4 (BMP4, Invitrogen, USA), the next day (Day 0). The medium was changed daily for 7 d to obtain hESC-Es. Differentiated hESC-Es at day 7 could be cultured with defined keratinocyte serum-free medium (DKSFM, Invitrogen, USA); changing the medium every second day, for another 1 week, or the cells were distributed onto gelatin-coated plates and split at a ratio of 1:3 in DKSFM to obtain confluent cell layers.

### Genomic DNA extraction and sequencing

Genomic DNA was extracted using the TIANamp Genomic DNA Kit (TIANGEN Biotech, Beijing, China). DNA fragments harbouring the 911-bp target sequence were amplified using the primer set PTCH1-MSD-F (TACTAATGAGCAGCAGGTAAGGCGG) and PTCH1-MSD-R (TGCTGGGGTGAAAACAGACAAGAGG), using KOD plus DNA polymerase (Toyobo, Japan), according to the manufacturer’s instructions. The touchdown PCR programme is shown in Table S1. Genomic DNA (150 ng) was used as the template for all reactions. The MSD-PCR product was electrophoresed on a 1% agarose gel and sequenced.

### Construction of Cas9/sgRNA and donor vector

sgRNA oligonucleotides were annealed and inserted into the pre-cut pCS vector (Biocytogen, Beijing, China) to obtain Cas9/sgRNA plasmids. The MSD-PCR product was purified and cloned into a pre-cut pUCA(Luc) vector (Biocytogen, Beijing, China) to obtain the pUCA(Luc)-target plasmid. Each Cas9/sgRNA plasmid was then transfected into HEK293T cells together with the pUCA(Luc)-target plasmid, and the cleavage activity of each sgRNA was tested using the Universal CRISPR Activity Assay Kit (Biocytogen, Beijing, China) with a GloMax 96 Microplate Luminometer (Promega, USA) in accordance with the manufacturer’s instructions. The pCS-positive plasmid, provided with the kit, was used as the positive control and a sample without sgRNA treatment was used as the negative control.

To construct the donor vector, the 873-bp fragment PTCH1-1 and the 473-bp fragment PTCH1-2, containing the c.403C>T mutation, were joined using overlap PCR to produce the 1.3-kb 5′ homologous arm fragment PTCH1-12, which was then purified and cloned into an intermediate vector, pL452 (Biocytogen, Beijing, China), containing a loxP-flanked PGK-puromycin resistance (PuroR) cassette. The 1-kb 3′ homologous arm fragment PTCH1-4 was amplified and cloned into the pUC57 plasmid (TIANGEN Biotech, Beijing, China) to obtain the vector pUC57-PTCH1-4. The PTCH1-12 PuroR fragment was then excised from the intermediate vector pL452 and assembled into the pUC57-PTCH1-4 vector to obtain the final targeting vector pUC57-PTCH1-HR. The plasmid profiles and primer sets used to construct the donor vector are shown in Fig. S2 and Table S2.

### Gene targeting and clonal selection

The donor vector and Cas9/sgRNA plasmids were prepared and purified using the EndoFree Maxi Plasmid Kit (TIANGEN Biotech, Beijing, China). hESCs cells were separated into single cells with TrypLE and were suspended in E8 medium containing a ROCK inhibitor at 10 μmol•L^-1^ before electroporation. Cells were washed with DPBS and resuspended in a resuspension buffer from the Neon Kit (Life Technologies, USA) at a final density of 1.0 × 10^7^ cells per mL. For each electroporation, 10 μg of mixed plasmids (5 μg donor vector and 5 μg Cas9/sgRNA plasmid) were added to 100 μL of buffered hESC cell suspension containing 1 × 10^6^ cells, and this was immediately electroporated at 1 100 V and 40 ms for 1 pulse using the Neon Transfection System (Life Technologies, USA). Transfected cells were then plated onto a Matrigel-coated 100-mm dish with E8 medium supplemented with a ROCK inhibitor at 10 μmol•L^-1^. The medium was changed to E8 medium only on the next day. Three days after electroporation, puromycin selection (0.2 μg•mL^-1^) was initiated, and this continued for several days. Resistant clones were picked and expanded in E8 medium with continuous drug selection. Surviving clones were verified by genomic PCR analysis and sequencing.

### Genomic PCR analysis and sequencing

Genomic PCR and sequencing were performed to screen positive clones. Mutation site PCR was performed using the primer pair PTCH1-F/PTCH1-R to screen clones with the c.403C>T heterozygous point mutation. Heterozygous double peaks were expected to occur when one allele was successfully mutated. Junction PCR was performed to screen puromycin-resistant clones. If the 5′ homologous arm and the loxP-flanked inverted puromycin-resistant cassette (PuroR) had successfully integrated into the hESC genome by homologous recombination, the primer sets PTCH1-5′-F (P1, in the *PTCH1* locus, upstream of the 5′ homologous arm) and PTCH1-5′-R (P2, in the PuroR cassette) would amplify a 1.5-kb product or would not generate a DNA product. If the PuroR cassette and the 3′ homologous arm had successfully integrated into the hESC genome by homologous recombination, the primer sets PTCH1-3′-F (P3, in the PuroR cassette) and PTCH1-3′-R (P4, in the *PTCH1* locus, downstream of the 3′ homologous arm) would amplify a 1.4-kb product or would not generate a DNA product. Long-range PCR using the primer set PTCH1-5′-F/PTCH1-3′-R (P1/P4) was performed to amplify a 4.4-kb and a 2.7-kb product to further identify integration of both homologous arms containing the PuroR cassette into one allele of the selected clones. All PCR amplifications were performed as mentioned previously (Table S1), and the primers used are listed in Table S3.

### Western blot analysis

Cells were lysed in RIPA buffer containing a protease inhibitor cocktail (Roche, Roswell, GA, USA) on ice for 30 min; samples were then centrifuged at 12 000 × *g* at 4 °C for 20 min to collect the supernatants. Protein concentrations were measured using the BCA Protein Assay Kit (Thermo Scientific, USA). Samples of 50 μg were separated by 8% SDS-PAGE and transferred to polyvinylidene difluoride (PVDF) membranes (Millipore, Billerica, USA). After blocking with 5% non-fat milk for 1 h at room temperature, the membranes were incubated overnight at 4 °C with primary antibodies, including rabbit anti-PTCH1 (1:1 000, Abcam, Cambridge, UK; catalogue No.: ab53715, detecting the N terminal of human PTCH1), anti-GLI1 (1:1 000, Cell Signaling Technology, Danvers, MA, USA) and mouse anti-GAPDH (1:5 000, ZSGB-BIO, Beijing, China), respectively. After three washes for 5 min each in TBST, membranes were incubated with anti-rabbit or anti-mouse horseradish peroxidase-conjugated secondary antibodies (Cell Signaling Technology, Danvers, MA, USA; diluted at 1:10 000) at room temperature for 1 h. Finally, the protein bands were visualized using the enhanced chemiluminescence method and an ECL detection kit (CWbiotech, Beijing, China). Band densities were analyzed by Gel-Pro Analyzer 4.0 software, and protein levels were normalized to those of GAPDH.

### Karyotype analysis

Cultured metaphase cells were prepared through treatment with 100 ng•mL^-1^ colcemid at 37 °C for 2 h, which was followed by hypotonic treatment in pre-warmed 0.075 mol•L^-1^ KCl. Cells were centrifuged and resuspended in freshly made fixative solution (methanol/glacial acetic acid, 3:1) and then dropped onto slides, dried and observed using a microscope.

### RNA extraction and real-time PCR analysis

Total RNA was extracted using TRIzol reagent (Invitrogen, USA). Reverse transcription into cDNA was performed with the RT Master Mix (TaKaRa, Osaka, Japan), according to the manufacturer’s protocol. Quantitative real-time PCR was performed using SYBR Green Master (Roche, Mannheim, Germany) with an ABI 7500 thermal cycler and the primers listed in Table 2. Relative gene expression was measured using the 2^−ΔΔCt^ method by normalizing to GAPDH expression levels.

**Table 2:** Primer sets used in real-time PCR

Primer	Sequence (5′- 3′)	Tm /℃	Product length/bp
GAPDH-F	GAGTCAACGGATTTGGTCGT	58.21	185
GAPDH-R	GACAAGCTTCCCGTTCTCAG	58.57	
K18-F	CCGTCTTGCTGCTGATGACT	60.39	200
K18-R	GGCCTTTTACTTCCTCTTCGTG	59.26	
ΔNP63-F	GGAAAACAATGCCCAGACTC	56.98	294
ΔNP63-R	GTGGAATACGTCCAGGTGGC	60.74	
NANOG-F	GCAGAAGGCCTCAGCACCTA	61.9	81
NANOG-R	AGGTTCCCAGTCGGGTTCA	60.46	
OCT4-F	GCTCGAGAAGGATGTGGTCC	60.18	81
OCT4-R	CGTTGTGCATAGTCGCTGCT	61.35	
PTCH1′-F	ACTTCAAGGGGTACGAGTATGT	58.56	113
PTCH1′-R	TGCGACACTCTGATGAACCAC	60.61	
GLI1-F	AGGGAGTGCAGCCAATACAG	59.75	171
GLI1-R	ATTGGCCGGAGTTGATGTAG	57.67	
SMO-F	GAAGTGCCCTTGGTTCGGA	59.93	212
SMO-R	GCAGGGTAGCGATTCGAGTT	60.18	
SHH-F	GCGAGATGTCTGCTGCTAGT	59.9	180
SHH-R	CCCTTCATACCTTCCGCTGG	60.18	

### Immunofluorescent staining

hESCs were seeded on Matrigel-coated glass slides in 12-well plates and induced for 7 d to obtain hESC-Es, as described previously. hESC-Es were fixed in 4% paraformaldehyde (Solarbio, Beijing, China) for 15 min, permeabilized with 0.25% TritonX-100 (Solarbio, Beijing, China) for 15 min, blocked with 5% bovine serum albumin for 1 h at room temperature and then incubated simultaneously with primary antibodies, including monoclonal rabbit anti-P63 and polyclonal mouse anti-K18 (1:500, Cell Signaling Technology, Danvers, MA, USA), overnight at 4 °C. Cells were stained with fluorophore-conjugated secondary antibodies, including Alexa fluor 488-conjugated anti-rat IgG (Cell Signaling Technology, Danvers, MA, USA) and rhodamine (TRITC)-conjugated anti-mouse IgG (ZSGB-BIO, Beijing, China), at 1:500 and 1:100 dilutions, respectively for 1 h at room temperature. The cells were mounted using mounting medium with DAPI (ZSGB-BIO, Beijing, China). Immunofluorescent images were observed using a laser scanning confocal microscope (Leica, Japan) with LAS AF Lite software.

### Cell proliferation assays

Cell proliferation was assessed using the CCK-8 assay (Dojindo, Japan). hESCs were seeded in 96-well plates (3 000 per well) and induced to form hESC-Es, as described previously. On day 7, the cells were treated with DMSO (Sigma, USA) or GDC-0449 (Selleck Chemicals, Houston, TX, USA) in DKSFM, changing the medium every other day for 1 week. The medium was changed to basic DMEM/F12 containing CCK-8 2 h before harvesting for the detection of OD values on days 0, 1, 3, 5, 7, 9, 11 and 13 using the CCK-8 kit according to the manufacturer’s protocol.

### Statistical analysis

Experiments were performed in triplicate. Data were analyzed with SPSS 13.0 (SPSS, Chicago, IL, USA) and were expressed as the mean ± standard deviation (SD). Student’s *t* tests and one-way ANOVAs were used to compare the differences between the groups. Values of *P* < 0.05 were considered statistically significant.

## Electronic supplementary material

Figure S1

Figure S2

Table S1

Table S2

Table S3

text summary-supplementary information
