# Supplementary figures and images for: Effect of the sonic hedgehog inhibitor GDC-0449 on an in vitro isogenic cellular model simulating odontogenic keratocysts
Source: Int J Oral Sci. 2019 Jan 5;11(1):4. doi: 10.1038/s41368-018-0034-x (PMC6320367; doi:10.1038/s41368-018-0034-x)

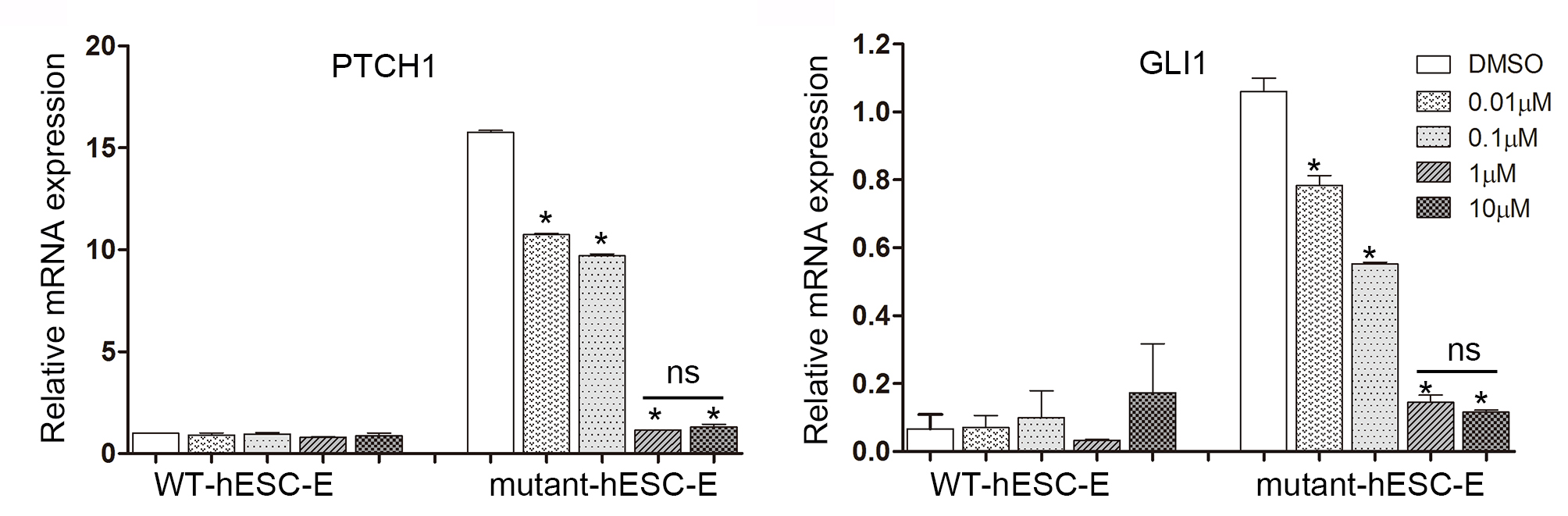

Supplement: Supplementary file 1 — Figure S1 [file 41368_2018_34_MOESM1_ESM.jpg]

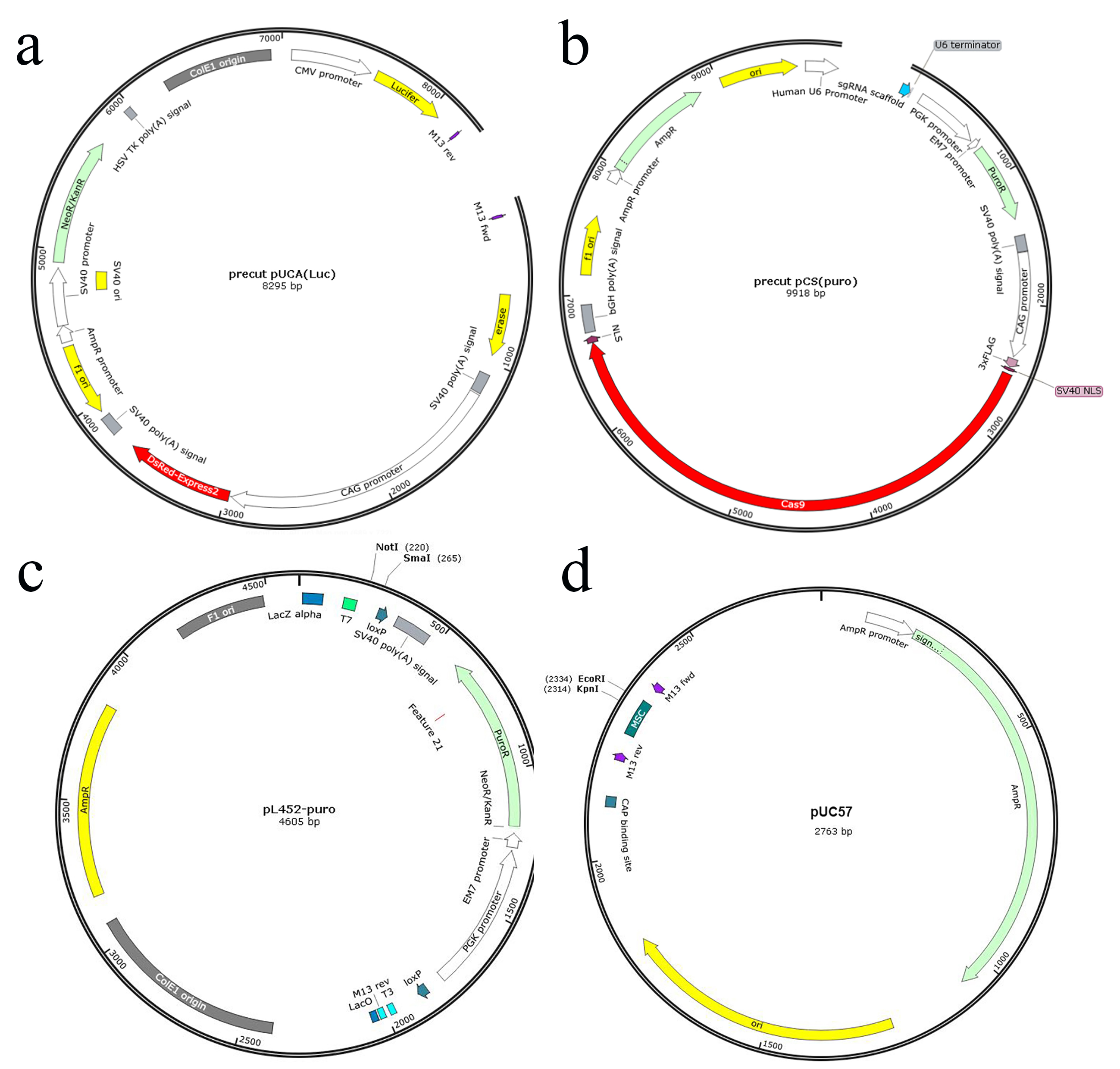

Supplement: Supplementary file 2 — Figure S2 [file 41368_2018_34_MOESM2_ESM.jpg]
