# Supplementary material for: Effect of the sonic hedgehog inhibitor GDC-0449 on an in vitro isogenic cellular model simulating odontogenic keratocysts
Source: Int J Oral Sci. 2019 Jan 5;11(1):4. doi: 10.1038/s41368-018-0034-x (PMC6320367; doi:10.1038/s41368-018-0034-x)
Supplement: Supplementary file 3 — Table S1 [file 41368_2018_34_MOESM3_ESM.docx]

| **Table S1. Touch-down PCR program to amplify genomic DNA** | | |
| --- | --- | --- |
| 94 °C | 5 min |  |
| 94 °C | 30 sec |  |
| 67 °C | 30 sec (-0.7 °C /cycle) | 15 cycles |
| 68 °C | 1 kb/min |  |
| 94 °C | 30 sec |  |
| 56 °C | 30 sec | 25 cycles |
| 68 °C | 1 kb/min |  |
| 68 °C | 10 min |  |
| 4 °C | forever |  |
