# Supplementary material for: Effect of the sonic hedgehog inhibitor GDC-0449 on an in vitro isogenic cellular model simulating odontogenic keratocysts
Source: Int J Oral Sci. 2019 Jan 5;11(1):4. doi: 10.1038/s41368-018-0034-x (PMC6320367; doi:10.1038/s41368-018-0034-x)
Supplement: Supplementary file 4 — Table S2 [file 41368_2018_34_MOESM4_ESM.docx]

| **Table S2. Primer sets used to construct donor vector** | | | |
| --- | --- | --- | --- |
| Primer | Sequence (5´- 3´) | Tm ( °C ) | Product name & length (bp) |
| PTCH1-1-F (NOTI) | *ctag***GC GGCCGC** ACAGTGTTGATGGTTTTCTTTGGTCAC | 60 | PTCH1-1 |
| PTCH1-1-R | ACGACTTACTC***A***TCCTCCAACTGACAAATATGTACAG | 63 | 873 |
|  |  |  |  |
| PTCH1-2-F | ATATTTGTCAGTTGGAGGA***T***GAGTAAGTCGTGAATTAAATTATACT | 62 | PTCH1-2 |
| PTCH1-2-R | TCCCCAAGTGGCTCAGCGTCATCCTCTTTT | 67 | 473 |
|  |  |  |  |
| PTCH1-1-F (NOTI) | *ctag***GC GGCCGC** ACAGTGTTGATGGTTTTCTTTGGTCAC | 60 | PTCH1-12 |
| PTCH1-2-R | TCCCCAAGTGGCTCAGCGTCATCCTCTTTT | 67 | 1304 |
|  |  |  |  |
| PTCH1-4-F(KPNI) | *ctag***GGTAC C** CTATACACCCAGGCTGTAAGATGTTTCA | 61 | PTCH1-4 |
| PTCH1-4-R(ECORI) | *ctag***G AATTC** TACGAGCACACATGACCATAAGCCC | 59 | 1063 |
| Bold capital letters represent restriction enzyme cutting sites. Italic lower case letters represent the sticky ends after annealing. Italic bold capital “A/T” represents the mutation point. | | | |
