# Supplementary material for: Effect of the sonic hedgehog inhibitor GDC-0449 on an in vitro isogenic cellular model simulating odontogenic keratocysts
Source: Int J Oral Sci. 2019 Jan 5;11(1):4. doi: 10.1038/s41368-018-0034-x (PMC6320367; doi:10.1038/s41368-018-0034-x)
Supplement: Supplementary file 5 — Table S3 [file 41368_2018_34_MOESM5_ESM.docx]

| **Table S3. Primer sets used to identify positive clones** | | | |
| --- | --- | --- | --- |
| Primer | Sequence (5´- 3´) | Tm | Product length (bp) |
|  |  | ( °C ) |  |
| PTCH1-F | GTTTGTGTCTGTGACAGGCGGGAC | 63 | 543 |
| PTCH1-R | TACTCCAGGTGCATTTCCAGGGCA | 63 |  |
|  |  |  |  |
| PTCH1-5'-F (P1) | GCTTCCTTTGCCCTAGACTGTGGAG | 62 | 1558 |
| PTCH1-5'-R (P2) | CGTCGACCTCTAGCGGATCCATAAC | 61 |  |
|  |  |  |  |
| PTCH1-3'-F (P3) | ATGCTCCAGACTGCCTTGGGAAAAG | 62 | 1426 |
| PTCH1-3'-R (P4) | AGGGATGCCACTTCACATTCTGCAA | 62 |  |
|  |  |  |  |
| PTCH1-5'-F (P1) | GCTTCCTTTGCCCTAGACTGTGGAG | 62 | 2700(WT) / 4393(KI) |
| PTCH1-3'-R (P4) | AGGGATGCCACTTCACATTCTGCAA | 62 |  |
| WT: wild type; KI: knocked-in mutation | | | |
