# Supplementary material for: Effect of the sonic hedgehog inhibitor GDC-0449 on an in vitro isogenic cellular model simulating odontogenic keratocysts
Source: Int J Oral Sci. 2019 Jan 5;11(1):4. doi: 10.1038/s41368-018-0034-x (PMC6320367; doi:10.1038/s41368-018-0034-x)
Supplement: Supplementary file 6 — text summary-supplementary information [file 41368_2018_34_MOESM6_ESM.docx]

**Figure S1, related to Figure 4. Effects of GDC-0449 on wild type (WT) and mutant hESC-Es in vitro.**

WT and mutant hESC-Es were treated with GDC-0449 or DMSO for 24 h. Gene expression was normalized to that of GAPDH. Data represent mean ± SD, n = 3 (*: GDC-0449 group vs. DMSO group in mutant hESC-Es. *: p < 0.05; ns: p > 0.05).

**Figure S2, Plasmid profiles.**

(a)Plasmid profile of precut pUCA(Luc). (b) Plasmid profile of precut pCS. (c) Plasmid profile of pL452-Puro. (d) Plasmid profile of pUC57.

**Table S1, Touch-down PCR program to amplify genomic DNA**

**Table S2, Primer sets used to construct donor vector**

**Table S3, Primer sets used to identify positive clones**
